# “Now I have to act on that”: Parent anticipation of a therapeutic odyssey following predictive testing for autism

**DOI:** 10.21203/rs.3.rs-7123259/v1

**Published:** 2025-09-08

**Authors:** Katherine E. MacDuffie, Aurora M. Washington, Catherine A. Burrows, Stephen R. Dager, Jed T. Elison, Annette M. Estes, Rebecca Grzadzinski, Chimei M. Lee, Joseph Piven, Mark D. Shen, Benjamin S. Wilfond, Jason Wolff, Lonnie Zwaigenbaum, John R. Pruett

**Affiliations:** Seattle Children’s Research Institute; Drake University; University of Minnesota; University of Washington; University of Minnesota; University of Washington; University of North Carolina at Chapel Hill; University of Minnesota; University of North Carolina at Chapel Hill; University of North Carolina at Chapel Hill; Seattle Children’s Research Institute; University of Minnesota; University of Alberta; Washington University School of Medicine in St. Louis

**Keywords:** autism, prediction, biomarker, susceptibility, bioethics, qualitative research, early intervention, infancy

## Abstract

**Background:**

Brain-based tools are being developed to identify infants at ultra-high likelihood for developing autism and enable presymptomatic intervention, though such interventions are not yet clinically available. Given persistent challenges in accessing autism services, we sought to understand how families might use early predictive results to seek support.

**Methods:**

We analyzed 55 interviews with parents of infants aged 6–13 months; one group had experience parenting an older autistic child (n = 30), the other had no prior autism parenting experience (n = 25). All parents were asked what steps they would take if told their infant was likely to develop autism.

**Results:**

Both groups described an intent to find appropriate services; parents with prior autism experience provided more specifics based on prior knowledge. The groups diverged in their anticipated supports and information sources. Parents with autism experience anticipated seeking financial support via insurance and disability benefits; those without autism experience reported they would consult their pediatrician for information or search online.

**Limitations:**

This qualitative study was conducted with a sample of parents selected for their specific life experiences, but likely does not capture the full range of potential responses to biomarker testing in infancy.

**Conclusions:**

Given that most services and benefits require a formal diagnosis, families receiving predictive results in infancy will likely face challenges finding appropriate services. Prior to implementing predictive testing in the first year of life, researchers should consider their obligation to support families who receive predictive results.

## Background

An autism spectrum disorder (hereafter autism) diagnosis can open many doors for families including granting eligibility for autism-specific interventions, insurance benefits, and general developmental services like speech, physical, and occupational therapy (1). The diagnosis, itself, is therefore one important step, but certainly not the end point, of the overall “therapeutic odyssey” that parents undertake to find the best therapies and supports for their child (2, 3). The odyssey metaphor conveys the great lengths and challenges parents must navigate to get their child connected to services, including researching options, getting on waitlists, negotiating complex paperwork and bureaucracy, and assessing the fit of a given therapy or approach for their specific child (4).

In the United States (US) the median age of autism diagnosis is 4 years (5), although most parents develop first concerns between ages 1–2 (6). Many new tools are being developed to detect susceptibility biomarkers^1^ even earlier in development (e.g., at 6–12 months; 8,9) that are strongly predictive of a future autism diagnosis and could be used to identify infants and toddlers who could benefit from presymptomatic intervention (10, 11). Some of these tools are designed for infant siblings of older children with autism, while others are intended to be applied in the general population. In addition to research advances, the US policy landscape is shifting towards accommodating more widespread clinical use of biomarker testing: 20 US states have passed legislation mandating some degree of insurance coverage for biomarker testing and similar bills have been proposed in an additional 13 states (12).

In a prior qualitative interview study we conducted with parents of infants with and without an older sibling with autism, almost all parents (54/55; 98%) reported that they would be interested in utilizing a biomarker-based predictive test if concerned about their infant’s development, with the primary motivations to enroll in earlier interventions, and have more time to prepare emotionally and financially (13). Here, with the same group of parents, we sought to gain understanding of the types of interventions and services parents anticipated they would seek after learning their infant was very likely to develop autism. Currently there are no autism-specific interventions clinically available for children age 6–12 months (14), and only one clinical trial has been published to date that includes children in this age range (15). In this context, we sought to understand the potential role of a positive biomarker result (i.e., a result predictive of future autism) in the therapeutic odyssey that families undertake. Learning how families expect to use a positive biomarker result is critical for identifying potential misconceptions about clinical utility which would need to be addressed in pre-test counselling alongside information about the risks, benefits, and accuracy of a particular testing modality (16). Furthermore, anticipating the additional support that families may need after biomarker testing can inform the design of future research studies that anticipate disclosing predictive results to families (17).

The three specific research questions guiding this analysis were: 1) What kinds of interventions do parents anticipate seeking following a positive biomarker result? 2) Where would parents turn to get additional information and support? 3) How do responses differ for parents with or without prior autism parenting experience?

## Methods

Fifty-five semi-structured phone interviews were conducted from January 2020 to January 2022 with two groups of parents: 30 had prior autism experience (AE) from parenting an older child with autism, and 25 had no autism experience (NAE), though most of the families in this group (20/25) had at least one older child. All AE and NAE parents had an infant who was in the age range targeted by many predictive tests: 6–13 months. Eligibility required having an infant in this age range and being able to participate in an interview in English. Study procedures for all AE parents were approved by the Washington University in St. Louis, and study procedures for NAE parents approved by the Seattle Children’s IRB. Informed consent for participation and audio recording was obtained verbally from all participants prior to beginning the interview, and all research procedures were performed in accordance with the United States Federal Policy for the Protection of Human Subjects (the Revised Common Rule). Participating parent demographics are presented in Table 1.

Separate semi-structured interview guides were developed by the authors and used to assess AE and NAE parent attitudes towards susceptibility biomarker testing for autism (full interview guides published elsewhere; see Washington et al., 2024). Interviewees were asked to imagine a potential predictive test (with predictive MRI offered as the primary example) that could tell between 6–12 months whether their infant was likely to develop autism (Table 2). The interviewer then posed a series of questions about parental interests and motivations for such testing and their anticipated response to a variety of potential test results. Interviews were conducted by phone by a licensed clinical psychologist and experienced qualitative interviewer and were audio-recorded and transcribed for analysis.

As a research team, we approached the interviews in an exploratory manner, with the goal of understanding how the complex, contextual, constructed, and subjective reality experienced by interviewees could be influenced by earlier knowledge that their child is (or is not) likely to develop autism (13,18). The multidisciplinary co-author team included researchers who focus on developing tools for autism prediction, clinicians, social scientists, and bioethicists, of varying ages, career stages, genders, and racial/ethnic identities. The interview guide questions were developed to describe technologies for autism prediction as neutrally as possible, with opportunities for in-depth exploration of interviewees’ perceptions of the potential benefits, risks, and anticipated impacts of employing predictive technologies for their infant.

We employed qualitative content analysis, a common analytic approach in health sciences research which focuses on the informational content of interviews (19). Deductive coding was conducted by two coders in Atlas.ti, with 70% of transcripts double coded and weekly meetings to resolve discrepancies. Additional details of the qualitative coding procedure can be found in our prior manuscript reporting on the overall interview findings (Washington et al., 2024). For the current analysis, we focused on a subset of the data: parental responses to being asked to imagine what next steps they would take in response to a positive biomarker result (Table 2).

## Results

### Anticipated interventions

When asked about specific next steps following a positive result, parents in both groups anticipated a period of emotional adjustment and then jumping into “action mode”:

“I think that I would probably like, right afterwards, I would take her home and just cuddle her and just like love on her a lot. And then pretty much like get to work.”

(NAE 3)

Parents described their role as primary advocates for their children to get access services following a positive result:

“There’s a huge burden of responsibility because as a parent, now I have to act on that and try to make my child’s path in life as easy as possible. ”

(NAE 24)

**Figure 1:**
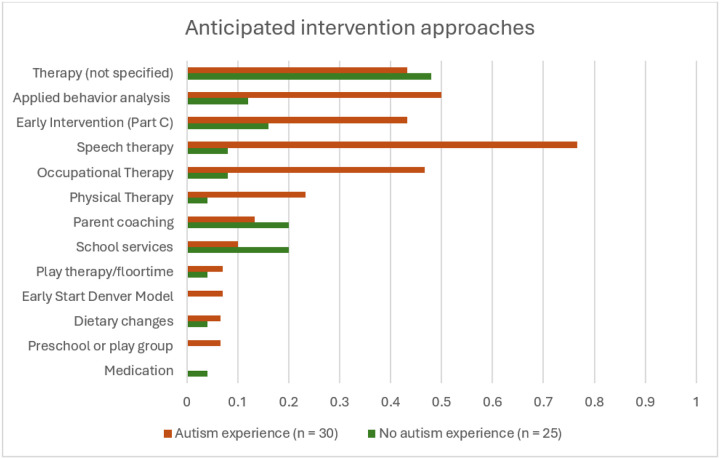
Anticipated intervention approaches. The proportion of parents in each group who anticipated engaging in each intervention type.

Parents in both groups described seeking therapies or supports for their infant (Fig. 1) However, AE parents were more likely to mention specific therapy types such as speech and language therapy, occupational therapy (OT), physical therapy, applied behavior analysis (ABA), and early intervention (i.e., in the USA, services funded through Part C of the Individuals with Disabilities Education Act). AE parents drew on their experience with their older child when describing their approach to intervention:

“With my son, he’s been receiving services. So naturalistic ABA, speech therapy, and OT. And I’ve seen how that’s really supported his development. So I would want to be able to offer the same to [infant], obviously, the earlier you start, the better.”

(AE 8)

NAE parents were less specific in their responses about anticipated interventions (i.e., did not name specific therapy modalities), but conveyed a similar intention to seek out any and all available interventions:

“But whatever, you know, programs or medicine or therapy that you can do for anyone for anything, we would probably hit it full bore. We would do whatever we could for him to make him thrive and you know, live a happy life.”

(NAE 6)

Other types of interventions mentioned by smaller proportions of parents included parent coaching, school services, dietary changes, play therapy, developmental preschool or play groups, and medication (see Fig. 1).

Parents in both groups (43% AE, 60% NAE) also anticipated changing how they interact with their child in response to a positive biomarker result, using words like “intentional” and “purposeful” to describe their approach. Many AE parents stated they were already using parenting strategies that they had learned with their older autistic child, but anticipated intensifying those efforts upon learning of their infant’s susceptibility:

“I think we would just accept it and then make sure that interaction with him was, you know, intentional and frequent…when [older child] was young, we read the Early Start Denver Model book.”

(AE 28)

NAE parents, in contrast, were again less specific about which parenting strategies they might employ, but conveyed similar intention as AE parents:

“He’s my fourth child. And I give him as much one on one time as I can. But I feel like if I were to know he had autism, I would probably work a lot harder on that and, you know, try to spend a lot more like engaged time with him.”

(NAE 4)

### Sources of other support or information

Beyond formal intervention, parents in both groups described where they might turn for additional support or information after receiving a positive biomarker result. The two groups diverged in the other sources of support they anticipated seeking following a positive result. AE parents, drawing on their prior experience, mentioned starting the process of getting connected with state or federal programs that provide autism-specific insurance or disability benefits for their infant. These types of financial supports were mentioned by almost a third of AE parents (9/30), and never mentioned by NAE parents (0/25), likely due to lack of familiarity. AE parents who described these supports saw clear advantages to learning about autism susceptibility earlier to begin the enrollment process:

“So with [older child], his ABA therapy, you can’t even get into it unless they are considered disabled through social security… so I mean, there’s a lot of things that you have to get your ducks in a row just to be able to get them the therapy. So I think it would be just starting all of that fun paperwork.”

(AE 24)

NAE parents, who were less familiar with insurance and disability benefits, were more likely to anticipate seeking social support. About a third of NAE parents mentioned seeking out support groups or finding other parents with similar experiences:

“You know, social parent groups we could join with other parents who are experiencing something similar…I’d probably want to talk to parents with children who are slightly older to learn, you know, what’s, what happened with them.”

(NAE 7)

**Figure 2:**
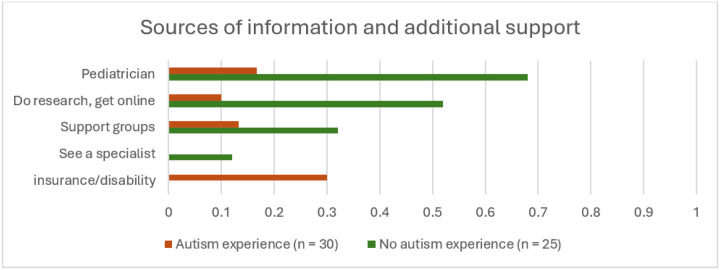
Anticipated sources of information and additional support. The proportion of parents in each group who anticipated seeking information or support from each source.

The two groups also diverged in their preferred information sources (Fig. 2). In contrast to parents in the AE group who were already knowledgeable about autism, over two thirds of parents in the NAE group anticipated turning to their pediatrician for information as a first step:

“I know their dad would definitely be all over the pediatricians, doctors, and he’d ask a lot of questions on what they can do to help.”

(NAE 1)

Many NAE parents also reported that they would do their “own research” and seek out information online, even as many acknowledged the risks of encountering overwhelming or potentially inaccurate information in online forums. As one parent put it:

“Probably I would get online. And I would say, you know, ‘living with an autistic child’, you know, ‘day to day, what to expect’ …I mean, one article would probably just lead to another, and it would just kind of spiral.”

(NAE 5)

### Questioning at what age interventions could (or should) start

Parents in both groups, though more commonly in the AE group, spontaneously questioned at what age interventions could start. Some conveyed an assumption that interventions for infants must be available:

“So I think that the whole point of the whole [predictive testing] thing is to be helpful. Like to figure out what types of doctors does my kid need to see, what types of treatments would they need to get? What should we be doing as parents to help them?

(NAE 16)

“I’m sure that if we could find out early on, we could start interventions early on, right?”

(AE 7)

Others actively questioned whether interventions were available for infants in the first year of life:

“I know about like ABA and Floortime and OT and stuff like that. But like, what does that look like for an eight-month-old?”

(AE 30)

“Is there any treatment at this time that would change the outcome?”

(NAE 12)

This uncertainty about interventions in this age range led some parents to anticipate they would need to seek information about the eligible age range for services from potential service providers:

“I think it would be reaching out to places and seeing like, okay, what’s the youngest that you have children come here with autism?”

(AE 21)

A few parents, recognizing this uncertainty, described the importance of providing support for parents in navigating services following a positive result.

“I think parents would have a hard time knowing what to do next. Because there isn’t a lot of information about like, you know, there isn’t, there hasn’t been diagnosis of a lot of infants.”

(NAE 2)

A small proportion of parents in both groups (20% AE, 8% NAE) felt like they might want to wait to start interventions for their infant until symptoms emerged, adding an additional element of uncertainty. Parents offered a few reasons for desire to wait. Some had bad prior experiences with early intervention or prenatal testing that led them to be more cautious. Others wanted to wait and see what symptoms would emerge for their infant to better target the treatment. As one parent put it:

“I feel like that would be another reason why you would wait until like, 12 or 18 months to start those therapies or interventions, you know, so you could, you know, make sure that, you know, this is what he needs.”

(AE 29)

## Discussion

When anticipating actions they would take in response to a positive biomarker result, parents with autism experience described navigating familiar paths to autism services, while parents without experience reported they would seek out information about available services and how to access them. AE parents were more likely to mention specific types of supports and parenting strategies, whereas NAE parents gave fewer specifics due to their lack of experience. The groups diverged in the sources they would turn to for additional information and support, with many AE parents describing seeking financial supports such as disability or insurance benefits, and many NAE parents, in contrast, describing turning to their pediatrician, internet sources, and/or support groups. Finally, some parents in both groups either conveyed an assumption that presymptomatic interventions were already available or actively questioned how early therapies could or should begin.

### Limitations

These results illuminate gaps in parental understanding and likely barriers to accessing services that could impact families in the near future as susceptibility biomarker testing moves towards clinical implementation. Importantly, however, they likely do not capture the full range of parental responses to biomarker testing in infancy, and the specific sample was selected because they had specific life experiences relevant to our research questions, but were not demographically representative of the general population (13). In addition, a limitation of this secondary analysis is that in the original semi-structured interviews, parents were asked broad questions about anticipated impacts (Table 2), rather than querying specific information sources or intervention types, which may have obscured or exaggerated differences between the two groups.

### Implications for autism biomarker research

The responses of parents in this study—both those naïve to and experienced with autism parenting—have important implications for the numerous ongoing efforts to develop tools for autism detection in the first year of life (10, 11). First, there is enormous variability across different jurisdictions in what “counts” as an autism diagnosis in order to qualify for autism-specific therapies (such as ABA) and insurance/disability benefits. Despite our intentional wording in the interview guide (Table 2), parents in many cases conflated a positive biomarker result with an autism diagnosis, and frequently assumed that a positive result would grant access to services. In reality, this would require systems-level change in eligibility criteria. Second, even if a positive biomarker result conferred eligibility, parents would likely still face challenges in finding appropriate services for their infant, embarking on a therapeutic odyssey that would be different (and less well-trod) than the path of parents seeking services for an older child with autism. Third and finally, there are real limitations to the sources of information that parents, particularly those naïve to autism, anticipate consulting following a positive result. Pediatricians have reported feeling unprepared to help families navigate the “black box” of autism care (20), and information found on the internet about autism is often inaccurate (21).

Taken together, these implications suggest that parents who undergo susceptibility biomarker testing will require additional supports/guidance. But who should provide this support? Given that many predictive tools remain in the research stage of development, investigators should consider their ancillary obligations to research participants (22). In anticipation of a therapeutic odyssey—at least while presymptomatic interventions are still being theorized, developed, and tested (14, 15)—consent conversations with families who are considering susceptibility biomarker testing should be transparent about the limitations of a presymptomatic biomarker result for conferring eligibility to the types of services families will seek. In the US, for example, even general early intervention services (Part C) require an established diagnosis or demonstrable developmental delay to qualify, and infants likely to develop autism may not yet have clinically-significant delays at 6–12 months (23). If researchers decide to share biomarker results with families, it can be argued they have a responsibility to either directly provide or assist families in finding appropriate services for their infants likely to develop autism (24). This could include connecting families to clinical trials of presymptomatic interventions (17). Clinical implementation of susceptibility biomarker testing should hinge upon the availability of autism-specific services and effective navigational supports for families, backed by evidence demonstrating that presymptomatic initiation improves child outcomes.

## Figures and Tables

**Table 1:** Demographics characteristics of parents

	Total (n=55)	Parents with autism experience (AE; n=30)	Parents with no autism experience (NAE; n=25)
Gender			
Female	52	29	23
Male	4	3	1
Non-Binary/ Queer	1	0	1
Race			
White	48	29	19
Black	3	2	1
Asian	4	0	4
Native American	1	0	1
Two or More	1	1	0
Ethnicity			
Hispanic	12	9	3
Non-Hispanic	45	23	22

Note: In two AE interviews, two parents participated. We have thus reported demographics for 32 parents who participated in 30 AE interviews. All NAE interviews were with individual parents.

**Table 2.** Introduction given to parents at the start of the interview and relevant questions in semi-structured interview guide

Interview introduction	We are interested in learning how parents in IBIS think about new methods in brain-imaging (like EEG and MRI) that are being developed to predict autism diagnoses very early, before the symptoms start. It is possible that predictive technology may be available in the next few years. We want to know what parents like you think about this possibility.
Introduction to relevant section of interview guide	Imagine that it were possible to use brain imaging at 6–12 months to predict which infants are likely to develop autism. I’d like you to imagine that right now we could tell you whether [CHILD NAME] is likely to develop autism.
Specific question	Imagine that you learned that [CHILD NAME] was likely to develop autism. What do you think your reaction would be?
Probes (i.e., follow-up questions)	How would if affect you? Your family? How would it affect your relationship with your child? Is there anything you might do or change as a result? [if not already mentioned] Would you seek out extra support or intervention? If so, what?

## Data Availability

Semi-structured interview guides have been previously published. The full codebook used for analysis is available from the corresponding author on reasonable request. Transcript and audio recordings are not publicly available to preserve participant privacy. De-identified copies of transcripts are available from the corresponding author on reasonable request.

## Footnotes

1. We use the term “susceptibility biomarker” in accordance with the definition developed by the NIHFDA Biomarker Working Group (7): “A biomarker that indicates the potential for developing a disease or medical condition in an individual who does not currently have clinically apparent disease or the medical condition.”

## Abbreviations

US: United States
AE: autism experience
NAE: no autism experience
OT: occupational therapy
ABA: applied behavior analysis
